# Streamlining Natural Products Biomanufacturing With Omics and Machine Learning Driven Microbial Engineering

**DOI:** 10.3389/fbioe.2020.608918

**Published:** 2020-12-21

**Authors:** Ahmad Bazli Ramzi, Syarul Nataqain Baharum, Hamidun Bunawan, Nigel S. Scrutton

**Affiliations:** ^1^Institute of Systems Biology (INBIOSIS), Universiti Kebangsaan Malaysia, Bangi, Malaysia; ^2^EPSRC/BBSRC Future Biomanufacturing Research Hub, BBSRC/EPSRC Synthetic Biology Research Centre, Manchester Institute of Biotechnology and School of Chemistry, The University of Manchester, Manchester, United Kingdom

**Keywords:** microbial engineering, synthetic biology, omics technology, machine learning, biomanufacturing, systems biology

## Abstract

Increasing demands for the supply of biopharmaceuticals have propelled the advancement of metabolic engineering and synthetic biology strategies for biomanufacturing of bioactive natural products. Using metabolically engineered microbes as the bioproduction hosts, a variety of natural products including terpenes, flavonoids, alkaloids, and cannabinoids have been synthesized through the construction and expression of known and newly found biosynthetic genes primarily from model and non-model plants. The employment of omics technology and machine learning (ML) platforms as high throughput analytical tools has been increasingly leveraged in promoting data-guided optimization of targeted biosynthetic pathways and enhancement of the microbial production capacity, thereby representing a critical debottlenecking approach in improving and streamlining natural products biomanufacturing. To this end, this mini review summarizes recent efforts that utilize omics platforms and ML tools in strain optimization and prototyping and discusses the beneficial uses of omics-enabled discovery of plant biosynthetic genes in the production of complex plant-based natural products by bioengineered microbes.

## Introduction

### Omics-Enabled Discovery of Plant Biosynthetic Genes

Plant natural products represent an enormous resource for chemical and biotechnological production of biopharmaceuticals and natural products-based drugs where about 50–70% of all anti-infective agents in clinical use are being provided and inspired by natural products (Newman and Cragg, 2016). As of 2019, up to 41.3% of anti-infective agents including antiviral and anti-malarial drugs were derived from natural products, which underlines the importance of these compounds as therapeutic agents (Newman and Cragg, 2020). With the advent of systems biology and omics research that have focused on investigating biological mechanisms at systems levels (Kitano, 2002; Lister et al., 2009), a plethora of bioactive compounds and relevant biosynthetic pathways has since been profiled and identified. This has culminated in the steady expansion of plant-based natural products datasets (Rai et al., 2017).

Driven by the increased availability of bioinformatics tools and high throughput instruments, including next generation sequencing (NGS) and mass spectrometry (MS), omics technologies have been prominently used as principal tools in systems biology research aimed at elucidating the underlying molecular mechanisms behind cellular functions and interplays among biomolecules in biological systems (Fridman and Pichersky, 2005; Sheth and Thaker, 2014; O’Brien et al., 2015). Omics technologies including DNA sequencing (genomics), RNA sequencing (RNA-seq; transcriptomics), and MS-based protein (proteomics) and metabolite (metabolomics) analyses have empowered the reconstruction of metabolic networks based on genome annotation and functional characterization of targeted biochemical reactions in a particular organism or system. The use of systems biology approaches in combination with computational methods has contributed to the generation of genome-scale metabolic models (GEMs) that are important in identifying all metabolic reactions and corresponding biosynthetic genes in various microbes and plants (Seaver et al., 2012; O’Brien et al., 2015).

Importantly, the adoption of single or multi-omics in natural products studies has seen the increment of omics-guided discovery of known and novel metabolites, biosynthetic genes, and regulatory elements from model and non-model plants. By employing transcriptome-guided gene mining and microbial engineering strategies, a number of natural products from previously incomplete and gapped pathways, such as opiate alkaloid noscapine and cannabinoids, have since been produced in microbial hosts, thereby opening up new and exciting opportunities in natural products biomanufacturing using bioengineered microbes as the preferred bioproduction platform (Li and Smolke, 2016; Luo et al., 2019; Courdavault et al., 2020). Biomanufacturing and commercialization of fermentation-based bioproducts, such as artemisinin, nootkatone, and β-farnesene, serve to demonstrate the feasibility and the bioeconomy potential of microbial engineering platforms in the production of fine chemicals and biopharmaceuticals (Benjamin et al., 2016; Ekas et al., 2019). In this mini review, recent applications of systems and synthetic biology approaches in the bioproduction of natural products are discussed where the advancement of natural products biomanufacturing using omics-driven microbial engineering and machine learning (ML)-assisted strain optimization strategies was further highlighted.

## Integration of Systems and Synthetic Biology for Microbial Production of Natural Products

Metabolic engineering and synthetic biology represent advanced bioproduction strategies that have allowed researchers to reprogram and modulate microbial metabolism using genetic and computational tools (Ramzi, 2018; Choi et al., 2019). Multi-omics approaches have been initially established for microbial systems leading to a growing number of reconstructed GEMs, especially in the universal chassis *Escherichia coli* and *Saccharomyces cerevisiae* where the computational sets of stoichiometric and mass-balanced metabolic reactions in the microbes were derived from genomics-guided experimental analysis including flux balance analysis (FBA) and elementary node analysis (Gu et al., 2019; Dahal et al., 2020). A host of systems biology, bioinformatics, and computer-aided design (CAD) tools has been developed and utilized to identify cellular metabolic bottleneck, pathway prediction, and gene design with the ultimate aim of enhancing bioproduction titers, rates, and yields (TRYs) by metabolically engineered microbes (Chae et al., 2017; Choi et al., 2019).

**FIGURE 1:**
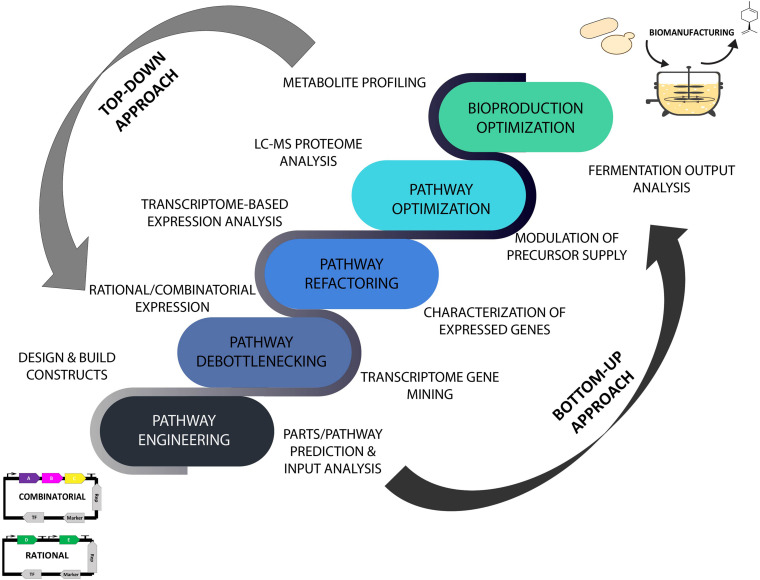
Top-down and bottom-up biomanufacturing strategies driven by omics and machine learning tools using high throughput analytical and predictive engineering technologies. Top-down approach focused on strain optimization and bioproduction improvement. Bottom-up approach aimed at reconstruction and refactoring of biosynthetic pathways for synthesis of existing and new-to-nature bioproducts. LC-MS, liquid chromatography–mass spectrometry.

The advent of data-driven systems and synthetic biology has brought a renewed and ever-increasing interest in translating laboratory strains into commercial-level microbial prototypes using omics- and *in silico*-guided biomanufacturing platforms that are expected to accelerate the scale-up process and speed up industrial scale production of desired products (Lee and Kim, 2015; Carbonell et al., 2018; Dunstan et al., 2020). The incorporation of the iterative Design-Build-Test-Learn (DBTL) cycle in microbial engineering approaches has provided a biological engineering and *in silico*-assisted framework for strain design and prototyping invaluable for industrial biotechnology applications. As part of the efforts in converging predictive analytics in improving bioproduction capabilities, the employment of metabolome, proteome, transcriptome, and bioinformatics analyses of the plant resources and microbial chassis has provided a comprehensive data-driven means for modulating and streamlining the biomanufacturing process of high-value natural products guided by the DBTL bioengineering framework (Casini et al., 2018; Carqueijeiro et al., 2020). An overview of data-guided bioproduction of natural products using systems and synthetic biology approaches is illustrated in Figure 1 where the implementation of omics technology and ML tools in improving top-down and bottom-up biomanufacturing strategies is further discussed in the following sections.

## Top-Down Approach: Omics-Guided Strain Design and Pathway Optimization

One of the key aspects of strain development using metabolic engineering and synthetic biology tools is the generation and characterization of biosynthetic genes as genetic parts in the pathway design of which the standardization in parts and plasmid assembly allows rapid strain prototyping *via* the DBTL iteration (Nielsen and Keasling, 2016; Robinson et al., 2020). In efforts to maximize TRYs of the natural products and precursor biosynthesis, omics-guided pathway analysis has been applied for a top-down microbial engineering approach by elucidating and identifying affected genes and proteins especially rate-limiting enzymes in engineered metabolic pathways (Table 1). In this top-down strain optimization approach, several omics platforms were employed in pathway debottlenecking and optimization in bioengineered microbial chassis that aimed at improving precursor supply and enhancing targeted natural product biosynthesis in a reverse engineering manner. With the focus on Test and Learn steps, proteome, metabolome, and bioinformatics analyses were conducted for the modulation of endogenous pathway intermediates, such as amino acids and isopentenyl pyrophosphate (IPP)-derived precursors, in bioengineered microbes. In particular, fine-tuning of IPP-related biosynthetic genes was found to be critical in optimizing terpenes bioproduction in engineered *E. coli* and *S. cerevisiae* owing to poor recombinant protein translation and precursor toxicity. Through proteome and transcriptome analyses of terpene-producing strains of *E. coli*, these pathway bottlenecks were debugged through codon optimization of the rate-limiting enzymes and the use of strong and regulated promoters, such as pTrc and pGadE (Redding-Johanson et al., 2011; Dahl et al., 2013). The application of principal component analysis of proteomics (PCAP) and multi-omics approaches in terpene-producing *E. coli* further demonstrated the importance of balanced and optimal protein expression, especially for HMG-CoA reductase, the key enzyme in the IPP-supplying mevalonate (MVA) pathway (Alonso-Gutierrez et al., 2015). In a seminal report by Brunk et al. (2016) on omics-guided microbial engineering, the combination of GEM, metabolomic, and proteomic analyses has allowed comprehensive pathway mapping and debottlenecking in MVA-derived terpene-overproducing *E. coli* by which several genes in the pentose phosphate pathway, tricarboxylic acid (TCA) cycle, and acetyl-CoA biosynthesis were found to be important in particular by downregulating pyruvate synthase (YDBK) gene that culminated in higher specific production of limonene. In genome engineered *S. cerevisiae*, the use of flux and metabolomic analysis has aided the functional expression of a heterologous 1-deoxy-D-xylulose 5-phosphate (DXP) pathway, the alternative IPP-producing pathway by which combinatorial expression of IspG (2-C-methyl-D-erythritol-2,4-cyclodiphosphate reductase) and IspH (4-hydroxyl-3-methylbut-2-enyl diphosphate reductase) enzymes was tested to overcome the poor conversion of 2-C-methyl-D-erythritol-2,4-cyclodiphosphate (MEcPP) and the limited NADPH coenzyme availability (Kirby et al., 2016).

**TABLE 1:** Omics-guided microbial engineering approaches for natural product and precursor biomanufacturing. Top-down approach mainly represented pathway debottlenecking and strain optimization for increasing bioproduction capacity. Bottom-up approach utilized transcriptome-enabled gene discovery for pathway engineering, refactoring, and bioproduction of industrially important natural products. MVA, mevalonate; PP, pentose-phosphate; TCA, tricarboxylic acid; DXP, 1-deoxy-D-xylulose 5-phosphate; BIA, benzylisoquinoline alkaloid.

Approach	Target metabolite (Chassis)	Key biosynthetic genes and parts	Omic-guided strategy	References
**Top-down**	**Terpene (*E. coli*)**	**MVA pathway** Mevalonate kinase (MK) and phosphomevalonate kinase (PMK) from *S. cerevisiae* under the control of *trc* promoter (*E. coli*)	Proteome-guided promoter characterization and pathway bottleneck debugging via codon optimization	Redding-Johanson et al., 2011
		**MVA pathway** Farnesyl pyrophosphate (FPP)-responsive promoters PybrL, PgadE, and PrstA controlling FPP biosynthetic genes	Promoter characterization and pathway intermediate toxicity measurement based on proteome and transcriptome dataset analysis of engineered *E. coli*	Dahl et al., 2013
		**MVA pathway** HMG-CoA synthase (HMGS) and HMG-CoA reductase (HMGR) from *Staphylococcus aureus*; Terpene synthase from *Mentha spicata* and *Abies grandis*	Application of principal component analysis for enzyme characterization and improvement based on proteome dataset of engineered *E. coli*	Alonso-Gutierrez et al., 2015
		**PP pathway** Phosphogluconate dehydrogenase (GND), glucose-6-phosphate dehydrogenase (G6PDH2r) in *S. cerevisiae* **TCA cycle** Isocitrate dehydrogenase (ICDHyr), alpha-ketoglutarate dehydrogenase (AKGDH) in *S. cerevisiae* **Acetyl-CoA biosynthesis** Pyruvate synthase (YDBK) in *S. cerevisiae*	Pathway precursor supply mapping using multi-omics (Metabolomics and proteomics) and GEM analysis	Brunk et al., 2016
	**Terpene precursor (*S. cerevisiae*)**	**MVA-associated pathway** HMG-CoA synthase (ERG13) and membrane protein (PRM10) in *S. cerevisiae* **DXP pathway** DXP biosynthetic genes (*Dxs, IspC, IspD, IspE, IspF, IspH*) from *E. coli*; 2-methyl-butenyl-4-diphosphate (HMBPP) synthase (IspG) from *E. coli, Bacillus subtilis, B. thuringiensis* and *Thermus thermophilus* **DXP-related redox system** Flavodoxin/ferredoxin NADP^+^-reductase (AtrFNR) from *Arabidopsis thaliana* and flavodoxin (Fld) from *E. coli* and *B. subtilis* **Iron-sulfur cluster (ISC) machinery** ISC operon (HscA, iscA, cyaY, iscS, iscU, hscB, fdx) and respiratory protein A (erpA) from *E. coli*	Genomics and metabolomics-assisted DXP pathway optimization	Kirby et al., 2016
	**Phenylpropanoid precursor (*S. cerevisiae*)**	**Coumaric acid biosynthesis** Tyrosine ammonia lyase (TAL) from *Flavobacterium johnsoniae*, shikimate kinase (aroL) from *E. coli*, tyrosine biosynthetic genes (m*ARO7*, m*ARO4, ARO10*) and pyruvate decarboxylase (PDC5) in *S. cerevisiae* **Amino acid and sugar transport** Tyrosine and tryptophan amino acid transporter (TAT1), polyamine transporter (TPO1), arginine transporter (ALP1), amino acids transporters (BAP2, AGP3), acetate transporter (ADY2) and galactose transporter (GAL2) in *S. cerevisiae*	Metabolic pathway characterization and optimization based on metabolomic and transcriptomic analysis	Rodriguez et al., 2017
		**Coumaric acid biosynthesis** Shikimate kinase (aroL) from *E. coli*, tyrosine ammonia-lyase (TAL) from *F. johnsoniae*, tyrosine biosynthetic genes (m*ARO7*, m*ARO4*, *ARO10*) and pyruvate decarboxylase (PDC5) from *S. cerevisiae* **PP pathway** Glucose-6-phosphate dehydrogenase (ZWF1), 6-phosphogluconolactonase (SOL3) and 6-phosphogluconate dehydrogenase (GND1) in *S. cerevisiae*	Transcriptome-guided metabolic pathway characterization and optimization	Borja et al., 2019
	**Flavonoid (*S. cerevisiae*)**	**Characterized promoters** pTDH1, pPGK1, pINO1, pSED1 and pCCW12 in *S. cerevisiae* **Naringenin biosynthesis** 4-coumarate:CoA ligase (Ps4CL) from *Petroselinum crispum*, CHS chalcone synthase from *Petunia x hybrida* (PhCHS) and CHI chalcone isomerase from *Medicago sativa* (MsCHI)	Promoter characterization and yield improvement via transcriptomic analysis	Gao et al., 2020
**Bottom-up**	**BIA (*S. cerevisiae*)**	**(*S*)-reticuline biosynthesis** Norcoclaurine synthase (PsNCS) from *Papaver somniferum*	Identification and functional expression of norcoclaurine synthase in *S. cerevisiae* for (*S*)-reticuline production from L-tyrosine	Xiao et al., 2013; DeLoache et al., 2015
		**(*R*)-reticuline biosynthesis** Reticuline epimerase (PsCYP82Y2) from *P. somniferum*	Identification and functional expression of reticuline epimerase in *S. cerevisiae* for conversion of (*S*) to (*R*)-reticuline	Desgagné-Penix et al., 2012; Farrow et al., 2015
		**Dihydrosanguinarine biosynthesis** 6-*O*-methyltransferase (6*O*MT), coclaurine *N*-methyltransferase (CNMT), 4′-*O*-methyltransferase 2 (4′*O*MT2), truncated berberine bridge enzyme (BBEΔN), cheilanthifoline synthase (PsCFS) and stylopine synthase (PsSPS), cytochrome P450 reductase (PsCPR), tetrahydroprotoberberine cis-*N*-methyltransferase (TNMT), (S)-cis-*N*-methylstylopine 14-hydroxylase (MSH) from *P. somniferum*	Transcriptome gene mining and expression of 10-gene pathway from *P. somniferum* for biosynthesis of dihydrosanguinarine in *S. cerevisiae*	Xiao et al., 2013; Fossati et al., 2014
		**Opioids biosynthesis** 1,2-dehydroreticuline synthase-1,2-dehydroreticuline reductase (DRS-DRR), salutaridine synthase (SalSyn), salutaridine reductase (SalR), salutaridinol 7-O-acetyltransferase (SalAT), thebaine 6-*O*-demethylase (T6*O*DM) from *P. somniferum*	Transcriptome gene mining, characterization and complete biosynthesis of opioids thebaine and hydrocodone in bioengineered *S. cerevisiae*	Xiao et al., 2013; Matasci et al., 2014; Galanie et al., 2015
	**Tropane alkaloids (*S. cerevisiae*)**	**Tropane alkaloid biosynthesis** *N*-methyl putrescine oxidase (MPO) from *Nicotiana tabacum*	Transcriptome gene mining, characterization and functional expression of putrescine oxidase in *S. cerevisiae*	Matasci et al., 2014; Srinivasan and Smolke, 2019
		**Tropane alkaloid biosynthesis** Polyketide synthase (AaPYKS), cytochrome p450 (AaP450), tropinone reductase (AaTRI, AaTRII) from *Anisodus acutangulus*	Transcriptome gene mining, characterization and functional expression of tropane alkaloid biosynthetic genes in bioengineered *S. cerevisiae*	Cui et al., 2015; Ping et al., 2019b
	**Tropane alkaloids precursor (*S. cerevisiae*)**	**Tropane alkaloid biosynthesis** Diamine oxidase (DAO) from *A. acutangulus*	Transcriptome gene mining, characterization and functional expression of diamine oxidase in *S. cerevisiae*	Cui et al., 2015; Ping et al., 2019a
	**Phenylpropanoid precursor (*S. cerevisiae*)**	**Phenylpropanoid biosynthesis** Cinnamate-4-hydroxylase (C4H) from *P. minus*	Transcriptome gene mining and expression of cinnamate-4-hydroxylase in *S. cerevisiae*	Loke et al., 2017; Ramzi et al., 2018
	**Breviscapine flavonoid (*S. cerevisiae*)**	**Breviscapine flavonoid biosynthesis** Flavonoid-7-*O*-glucuronosyltransferase (F7GAT) and flavone-6-hydroxylase (F6H) from *E. breviscapus*	Transcriptome gene mining, characterization and reconstitution of complete breviscapine flavonoid pathway from *E. breviscapus* in bioengineered *S. cerevisiae*	Liu et al., 2018
	**Triterpenoid saponin (*S. cerevisiae*)**	**Triterpenoid saponin biosynthesis** Cucurbitadienol synthase (SgCDS), epoxide hydrolase (SgEPH3EPH), cytochrome p450 (SgCYP87D18) from *Siraitia grosvenorii*	Production of mogroside V compounds by bioengineered *S. cerevisiae* expressing *S. grosvenorii* enzymes	Tang et al., 2011; Itkin et al., 2016
	**Cannabinoids (*S. cerevisiae*)**	**Cannabinoids biosynthesis** Prenyltransferases (CsPT), tetraketide synthase (*C. sativa* TKS; CsTKS), olivetolic acid cyclase (CsOAC), acyl activating enzyme (AAE). Cannabinoid synthases THCAS and CBDAS from *C. sativa*	Transcriptome gene mining, characterization and reconstitution of cannabinoid biosynthetic pathway in bioengineered *S. cerevisiae*	van Bakel et al., 2011; Luo et al., 2019

Omics tools have also been utilized in elucidating cellular changes in yeast chassis engineered to produce aromatic phenylpropanoids *via* the shikimate pathway using aromatic amino acid L-phenylalanine or L-tyrosine as the main entry routes for phenylpropanoid biosynthesis. Metabolomic and transcriptomic analyses of p-coumaric acid (p-CA)-overproducing *S. cerevisiae* revealed distinct transcriptional changes of genes related to sugars and amino acids transport in S288c and CEN.PK background strains that aided in the efforts of systematically modulating the final production of p-CA with up to 20–50% improvement (Rodriguez et al., 2017). Further transcriptome-guided pathway optimization enabled enhanced p-CA bioproduction from xylose in which deletion of the tyrosine and tryptophan amino acid transporter TAT1 resulted in 50% increased of the p-CA titer (Borja et al., 2019). Similar transcriptome-assisted bioengineering strategies were employed to build and test multiple sets of yeast promoters including pINO1, pSED1, and pCCW12 that conferred increased naringenin production from p-CA in engineered *S. cerevisiae* (Gao et al., 2020). Evidently, the application of omic technologies in chassis optimization, especially in the Test and Learn synthetic biology cycle, is inordinately advantageous in pathway debottlenecking and increasing the TRYs of the desired natural products.

## Bottom-Up Approach: Omics-Enabled Pathway Engineering and Refactoring for Natural Products Biomanufacturing

The employment of single- and multi-omic tools has brought about a systematic biology-informed pipeline for discovering and biomanufacturing of new-to-nature plant-derived compounds using systems and synthetic biology platforms (Goh, 2018; Chen et al., 2020; Jamil et al., 2020). In the Design and Build steps, genes involved in plant and microbial natural products pathways are considered as important genetic parts by which reconstruction and combinatorial expression of the corresponding biosynthetic pathways have yielded a plethora of industrially important natural products and biochemicals in bioengineered microbes. Discovery of key and missing enzymes in plant biosynthetic pathways has been greatly expedited with transcriptome gene mining of non-model plants and expression of the candidate genes in microbial systems (Goh et al., 2018; Ku Bahaudin et al., 2018; Pyne et al., 2019). Two alkaloid-enriched plants specifically *Papaver somniferum* (opium poppy) and *Catharanthus roseus* (Madagascar periwinkle) have emerged as the model medicinal plants with regard to the employment of multi-omics approaches in the comprehensive analysis of the benzylisoquinoline alkaloids (BIAs) and monoterpenoid indole alkaloids (MIAs) biosynthetic pathways, respectively (Facchini and De Luca, 2008; Scossa et al., 2018). Using multi-omics strategies, the complete biosynthetic pathway of the anticancer drug vinblastine in *C. roseus* has been finally elucidated where a total of 31 steps are required for MIA compound synthesis from geranyl pyrophosphate (GPP) where the key redox and hydrolase enzymes for the conversion of stemmadenine to tabersonine or catharanthine were successfully identified *via* proteome analysis and transcriptome gene mining (Caputi et al., 2018). These omics-driven strategies were similarly employed for the identification and expression of terpene and phenylpropanoid biosynthetic genes from the aromatic plant *Polygonum minus* (*Persicaria minor*) essential for pathway reconstruction and natural product biosynthesis in engineered microbes (Ramzi et al., 2018; Rusdi et al., 2018; Tan et al., 2018).

### Transcriptomic-Driven Design and Build of High-Value Natural Products in Microbial Chassis

One of the prominent examples of omics-enabled discovery and production of high-value natural products is the bioproduction of BIAs where candidate genes were obtained from the transcriptome datasets of BIA-accumulating plants, thereby representing a bottom-up approach in natural products biomanufacturing. The production of (*S*)- and (*R*)-reticuline was first demonstrated in engineered *S. cerevisiae* through BIA pathway reconstitution that includes the expression of the enzymes norcoclaurine synthase (NCS) and reticuline epimerase (CYP82Y2) from opium poppy *P. somniferum* (DeLoache et al., 2015; Farrow et al., 2015; Table 1). Through gene mining of *P. somniferum* transcriptome datasets, microbial expression of long and complex pathway of BIAs allowed the bioproduction of bioactive dihydrosanguinarine, thebaine, and hydrocodone compounds in engineered *S. cerevisiae* (Fossati et al., 2014; Galanie et al., 2015). Reconstruction and implantation of plant biosynthetic pathways can be modulated and programmed to exploit intrinsic amino acid pathways, such as L-phenylalanine, L-tryptophan, and L-ornithine, thereby removing the metabolic barriers for precursor and energy supply. Combinatorial and rational design strategies have enabled the biosynthesis of tropane alkaloids where *de novo* production of *N*-methylpyrrolinium, tropine, and cinnamoyl tropine has been attained through the incorporation and conversion of L-ornithine- and L-phenylalanine-derived intermediates, respectively, through the expression of corresponding *N*-methyl putrescine oxidase (MPO) from *Nicotiana tabacum* and tropane alkaloid biosynthesis genes from *Anisodus acutangulus* (Ping et al., 2019a, b; Srinivasan and Smolke, 2019).

Transcriptome analysis of antioxidant-rich medicinal plants, including *P*. *minus* and *Erigeron breviscapus*, revealed the candidate biosynthetic genes for phenylpropanoid-derived flavonoids and breviscapine that shared L-phenylalanine as the main intermediate compound in the plant biosynthetic pathway (Loke et al., 2017; Liu et al., 2018). The introduction of key biosynthetic genes, such as cinnamate-4-hydroxylase (C4H), flavone-6-hydroxylase (F6H), and flavonoid-7-*O*-glucuronosyltransferase (F7GAT), enabled pathway reconstruction and directed biosynthesis of the desired phenylpropanoid compounds in engineered *S. cerevisiae* using glucose as carbon source (Liu et al., 2018; Ramzi et al., 2018). Interestingly, the presence of endogenous MVA and squalene biosynthetic pathways in *S. cerevisiae* serves as a starting platform for transcriptome-enabled biosynthesis of cannabinoids and triterpenoid saponin that were naturally derived from *Cannabis sativa* L. and *Siraitia grosvenorii*, respectively. Complete biosynthesis of cannabinoids was demonstrated through the expression of *Cannabis* enzymes that include newly identified *Cannabis* candidate prenyltransferases that are responsible for the conversion of olivetolic acid and GPP supplied by native MVA and heterologous hexanoyl-CoA biosynthetic pathways, respectively (Luo et al., 2019). Using a MVA-dependent squalene pathway in *S. cerevisiae*, the biosynthesis of triterpenoid mogrol compounds was achieved *via* pathway reconstitution and heterologous expression of cucurbitadienol synthase, epoxide hydrolase, and cytochrome p450 identified from *S. grosvenorii* transcriptome (Itkin et al., 2016). Overall, the utilization and expression of transcriptome-derived plant biosynthetic genes represent an increasingly valuable and feasible strategy in pathway engineering and natural product biomanufacturing using bioengineered microbes as cell factories.

## The Way Forward: Streamlining Natural Products Biomanufacturing With Omics and ML Platforms

To date, model microbes, especially *E. coli* and *S. cerevisiae*, represent the most suitable natural product chassis for strain improvement and biological engineering using DBTL iteration and upscaling processes owing to increased availability of genetic parts and biological data, including GEMs and omics datasets. As discussed earlier, omics technologies have been valuable in enhancing synthetic biology applications, but progress remains in accelerating the Learn step needed to inform the next Design phase and consequent DBTL cycles important in improving the desirable specification and biomanufacturing capacities. Recent progress in advanced genomics and synthetic biology has seen the increased adoption of ML-based data training and non-biased predictive tools for analyzing biological datasets to complement the biology-informed systems biology approaches. The predictive ability of ML tools is empowered through training and learning of experimental data *via* statistical linkage and modeling of independent and dependent variables as input and output data, respectively (Radivojević et al., 2020). Critically, the employment of ML approaches in strain design and optimization is gaining much interest, which is expected to address limitations in biology-informed approaches and circumvent the needs for detailed mechanistic understanding and resource constraints (Carbonell et al., 2019; Presnell and Alper, 2019).

### Improving DBTL Performance and Predictive Capacities With ML and Omics Tools

ML-based training of biological datasets has been successfully used in microbial hosts in the efforts to improve gene annotation, metabolic pathway optimization, and fermentation bioprocess parameters (Kim et al., 2020). The bioproduction of specialty and fine chemicals, such as dodecanol and limonene, has been demonstrated in engineered *E. coli* and *S. cerevisiae* using ML-generated predictive models, which enabled unbiased genetic designs and combination (Zhou et al., 2018; Jervis et al., 2019; Opgenorth et al., 2019). A key advantage of utilizing ML tools is the development of a pure *in silico* system applicable for the Design and Learn phases that enable the selection of high-performing biological system without the needs to perform extensive and costly *in vivo* screening experiments. In the Design step, several ML tools have been developed for optimizing gene expression and cellular protein synthesis through *de novo* and quantitative design of genetic parts including promoter, 5′-untranslated region (5′UTR), and ribosomal binding site (RBS) in addition to the use of ML-assisted directed evolution and semi-rational protein engineering strategies (Decoene et al., 2018; Jervis et al., 2019; Wu et al., 2019). By training of partial least square (PLS) regression model on fluorescence output of a yeast UTR (yUTR) library, a newly constructed yUTR calculator was used to accurately predict the outcome of translation initiation rates in *S. cerevisiae* (Decoene et al., 2018). Employment of the predictive yUTR calculator enabled a tailored *in vivo* p-CA production in tyrosine ammonia lyase (TAL1)-expressing *S. cerevisiae* in accordance to the strengths of *de novo* and native 5′UTR with weak and high predicted protein abundance (Decoene et al., 2018).

Modulation and improvement of terpenes production has also been demonstrated through ML-enabled fine-tuning of gene expression by synthetic promoters and RBS of the MVA and non-MVA DXP biosynthetic genes (Meng et al., 2013; Jervis et al., 2019). Using a mutated Trc promoter and RBS sequences for artificial neural network (NN)-based model training and test, the expression of 1-deoxy-D-xylulose-5-phosphate synthase (DXS) gene under the control low-strength synthetic s14 promoter enhanced the production of amorphadiene in engineered *E. coli* (Meng et al., 2013). A recent report (Jervis et al., 2019) has expanded the use of a feedforward NN-based ML model on *de novo* design and screening of synthetic RBS for MVA pathway engineering and bioproduction of limonene where model training was conducted on expression levels of HMG-CoA synthase (HMGS), HMG-CoA reductase (HMGR), MVA kinase (MK), and IPP delta-isomerase (IDI) using multiple combinations of RBS sequences. The constructed library of 32 RBS combinations was then built and tested in combination with terpene-synthesizing pGL403 plasmid construct that resulted in the identification and selection of high-performing *E. coli* strains with improved limonene titer over 1.5–3-fold (Jervis et al., 2019).

The combination of omics datasets and ML strategies is expected to drive the production of natural products and other biobased chemicals especially in terms of biosynthetic pathway inference, refactoring, and optimization. The self-organizing map (SOM) approach represents an unsupervised NN method useful in the identification of new enzymes using plant transcriptome datasets to complement common gene co-expression analysis, such as differentially expressed genes (DEG) method (Dugé de Bernonville et al., 2020). The SOM-assisted co-expression analysis of *Rauvolfia serpentina* transcriptome has led to the identification of sarpagan bridge enzyme (SBE) and vinorine hydroxylase (VH) essential in sarpagan and ajmalan alkaloid biosynthesis that could be useful in the Build and Test of these high-value bioproducts in engineered microbial chassis (Dang et al., 2017, 2018). A supervised ML platform has been developed and tested using proteome and metabolome datasets of biofuel- and terpene-producing *E. coli* where the ML-driven model predictions yielded an accurate *in silico* pathway design and outperformed classical Michaelis–Menten kinetic modeling (Costello and Martin, 2018). In their report, a Tree-based Pipeline Optimization Tool (TPOT) was used for training data and succeeded in generating models for dynamically predicting medium level limonene-producing *E. coli* strains using experimental omics datasets, thus providing a pure ML and omic dataset-based virtual strain simulation and pathway construction (Costello and Martin, 2018). Interestingly, another recent report by Radivojević et al. (2020) leveraged on ensemble approach and probabilistic modeling methods to construct a ML-based Automated Recommendation Tool (ART) useful for improving microbial engineering and DBTL bioproduction performance by training of proteome datasets among a host of experimental data as input variables. By comparing limonene bioproduction improvement in engineered *E. coli* guided by experimentally tested PCAP, the ML models generated by ART were suggested to be able to match and further enhance the production of a given product through the DBTL cycle by recommending new inputs, such as transcriptome datasets and promoter strengths in the next Design phase. Following this, the integration of transcriptome, proteome, and/or metabolome datasets with ML methods is particularly useful in the development of mathematical models in the Test and Learn cycle that would guide and facilitate *in silico* optimization of the DBTL pipeline (Presnell and Alper, 2019; St. John and Bomble, 2019; Volk et al., 2020). Thanks to the growing list of genome, transcriptome, and GEM resources, further adoption and implementation of *in silico* and ML tools on these biological datasets are expected to bring about a markedly improved and accurate predictive engineering and retrosynthetic design of metabolic pathways to existing and new-to-nature chemicals (Lin et al., 2019; Zhang et al., 2020). In line with the emergence of data-driven 4^th^ Industrial Revolution (4IR), the applications of omics and ML tools in strain and bioproduct development are set to be the cornerstone in industrial biomanufacturing of biobased chemicals and pharmaceuticals.

## Conclusion and Perspectives

Overall, it is envisioned that the employment of data-centered omics and ML platforms will lead to more streamlined and less resource-intensive biomanufacturing strategies and accelerate strain prototyping pipelines that have been a major stumbling block in the translation of bioproduct development from laboratory to market. Omics-guided microbial engineering and ML-assisted biomanufacturing will therefore bring about data-driven biomanufacturing pipelines that can be expanded to include metagenome datasets and accelerate the bioproduction of industrially relevant biomolecules and drugs tailored to the pressing needs of medical, agricultural, environmental, and industrial sectors.

## Author Contributions

ABR, SNB, HB, and NSS all contributed toward the writing and editing of this manuscript. All authors contributed to the article and approved the submitted version.

## Conflict of Interest

The authors declare that the research was conducted in the absence of any commercial or financial relationships that could be construed as a potential conflict of interest.
